# Autocrine Human Urotensin II Enhances Macrophage-Derived Foam Cell Formation in Transgenic Rabbits

**DOI:** 10.1155/2015/843959

**Published:** 2015-11-12

**Authors:** Sihai Zhao, Yafeng Li, Shoucui Gao, Xiaojing Wang, Lijing Sun, Daxing Cheng, Liang Bai, Hua Guan, Rong Wang, Jianglin Fan, Enqi Liu

**Affiliations:** ^1^Laboratory for Lipid Metabolism and Atherosclerosis, Xi'an Jiaotong University Cardiovascular Research Center, Xi'an, Shaanxi 710061, China; ^2^Laboratory Animal Center, Xi'an Jiaotong University School of Medicine, Xi'an, Shaanxi 710061, China; ^3^Department of Molecular Pathology, Interdisciplinary Graduate School of Medicine and Engineering, University of Yamanashi, Yamanashi 409-3898, Japan

## Abstract

Circulating urotensin II (UII) is involved in the development of atherosclerosis. However, the role of autocrine UII in the development of atherosclerosis remains unclear. Here, we tested the hypothesis that autocrine UII would promote atherosclerosis. Transgenic rabbits were created as a model to study macrophage-specific expressing human UII (hUII) and used to investigate the role of autocrine UII in the development of atherosclerosis. Transgenic rabbits and their nontransgenic littermates were fed a high cholesterol diet to induce atherosclerosis. Comparing the transgenic rabbits with their nontransgenic littermates, it was observed that hUII expression increased the macrophage-positive area in the atherosclerotic lesions by 45% and the positive area ratio by 56% in the transgenic rabbits. Autocrine hUII significantly decreased the smooth muscle cell-positive area ratio in transgenic rabbits (by 54%), without affecting the plasma levels of total cholesterol, triglycerides, low-density lipoprotein cholesterol, high-density lipoprotein cholesterol, and glucose and adipose tissue contents. These results elucidated for the first time that autocrine UII plays an important role in the development of atherosclerosis by increasing the accumulation of macrophage-derived foam cell.

## 1. Introduction

Atherosclerotic cardiovascular diseases, including heart attack, stroke, and peripheral vascular insufficiency, continue to be the principal cause of death and disability in the world. Vasoactive peptides play an important role in the development of atherosclerosis [1]. Urotensin II (UII) is a vasoactive cyclic peptide composed of 11 amino acids, which was initially isolated from the neurosecretory system of goby fish [2, 3]. UII was expressed predominantly in the cardiovascular and central nervous systems as well as in endocrine tissue and, particularly, in human atherosclerotic lesions [4, 5]. Epidemiological, clinical, and experimental studies have shown that an increasing level of circulating UII is involved in the development of atherosclerosis [5–10]. Circulating UII increases plasma reactive oxygen species (ROS) and oxidized low-density lipoprotein (ox-LDL) and upregulates the expression of vascular cell adhesion protein-1 (VCAM-1), intercellular adhesion molecule-1 (ICAM-1), scavenger receptors (CD36 and scavenger receptor class A), and acyl-CoA, which are important molecules in the initiation and progression stages of atherosclerosis lesion formation [8–10]. Whether autocrine UII plays an important role in the development of atherosclerosis remains unclear. Macrophage-derived foam cells is one of the main cell components in atherosclerosis lesions and its differentiation, proliferation, and accumulation affect atherosclerosis plaque formation, progression, and destabilization [11]. We hypothesized that autocrine UII might promote atherosclerosis. In this study, we created transgenic rabbits to investigate the role of autocrine UII in the development of atherosclerosis by the macrophage-specific expression of human UII. Our results showed that macrophage-autocrine UII increased the accumulation of macrophage-derived foam cells in arch atherosclerotic plaques.

## 2. Materials and Methods

### 2.1. Generation and Identification of Human UII Transgenic Rabbits

**Figure 1:**
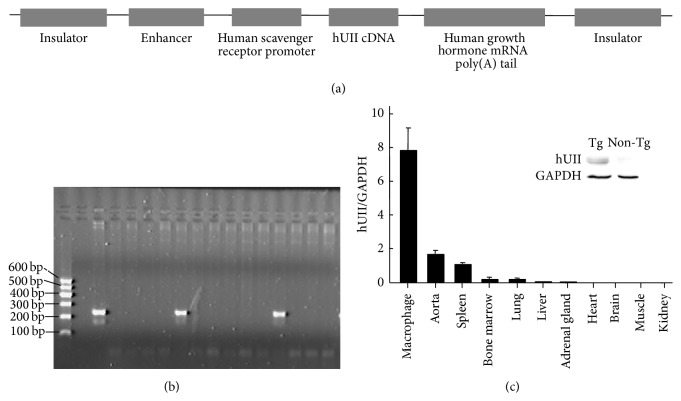
The generation and identification of the hUII transgenic rabbits. (a) The macrophage-specific transgenic construct for the microinjections. (b) The identification of the hUII gene integration by PCR in the rabbits (M, DNA marker; −, negative control; +, positive control plasmid; 1–13, rabbits DNA sample). (c) The real-time PCR and Western blotting analysis of hUII in alveolar macrophages. Tg, transgenic; M*φ*, macrophage.

Japanese white rabbits were supplied by the Laboratory Animal Center of Xi'an Jiaotong University. The generation of the transgenic rabbits expressing human UII (hUII) was conducted in our laboratory by microinjection, as described in a previous study [12]. For the macrophage-specific expression of hUII, 648 bp cDNA of the (NM_021995) hUII gene was cloned into EcoRV and SacII sites 3′ of the human scavenger receptor promoter and 5′ of the human growth hormone splicing and polyadenylation sites, with 4 copies of the chicken b globin insulator, which could prevent the position effect of transgenes [13]. The resultant fragment (Figure 1(a)) was isolated by digestion with Sal I, injected into fertilized rabbit zygotes, and reimplanted into foster mothers. The transgenic founders were identified from blood DNA by the polymerase chain reaction (PCR) method (primers: forward 5′TTCATCTTATGCTCTGCGTCAC 3′, reverse 5′ CTGGCAGTATCTGTAGAAGGGA 3′, 201 bp). The transgenic rabbits that had incorporated the transgene into the germ line were bred with nontransgenic rabbits. The hUII transgenic rabbits and their nontransgenic littermates were fed a high cholesterol diet and underwent atherosclerosis analysis. The animal experiments were approved by the Laboratory Animal Administration Committee of Xi'an Jiaotong University and performed according to the Guidelines for Animal Experimentation of Xi'an Jiaotong University and the Guide for the Care and Use of Laboratory Animals published by the US National Institutes of Health (NIH, Publication Number 85–23, revised 2011).

### 2.2. Isolation and Analysis of RNA

Peritoneal macrophages were harvested from the peritoneal cavity 4 days after injection of 4% Fluid's thioglycolate medium. Alveolar macrophages were collected by subjecting the lungs to lavage with 100 mL of phosphate buffered saline (PBS, pH 7.4). Peritoneal and alveolar macrophages were confirmed based on the criteria of morphology and nonspecific esterase reactivity. The total RNA from various tissues and isolated peritoneal and alveolar macrophages was rapidly isolated using TRIzol reagent (Invitrogen, USA) and reverse-transcribed into cDNA using a reverse transcription kit (Takara, Japan). The expression of hUII in the macrophages, heart, lung, liver, spleen, kidney, and small intestine was evaluated using quantitative real-time PCR. The following sets of primers were used: forward 5′ TTCATCTTATGCTCTGCGTCACTT 3′, reverse 5′ATGTTGGTACTTGAGTCTGCTTTCC3′, 259 bp; rabbit GAPDH forward 5′ATCACTGCCACCCAGAAGAC3′, reverse 5′ GTGAGTTTCCCGTTCAGCTC 3′, 146 bp. The cycling conditions were 95°C for 30 s, followed by 40 cycles of 95°C for 30 s, and 55°C for 40 s.

### 2.3. Western Blot Analysis of hUII

To identify the hUII expression levels in transgenic rabbits, protein samples extracted from alveolar macrophages of the transgenic rabbits and their nontransgenic littermates were prepared, as described previously, and analyzed by electrophoresis on 10% SDS-polyacrylamide gels, followed by Western blotting and probing with a polyclonal antibody against hUII (Atlas Antibodies, Sweden) [10].

### 2.4. Induction of Atherosclerosis

The hUII transgenic rabbits (*n* = 5, male) and their nontransgenic littermates (*n* = 5, male) were fed a high cholesterol diet (HCD, 0.3% cholesterol) for 16 weeks to induce atherosclerosis.

### 2.5. Measurement of Plasma Parameters

After overnight fasting, blood samples were collected via the auricular artery every two weeks. The blood samples were stored on ice and centrifuged (3000 rpm, 15 minutes, 4°C) to obtain the plasma. The plasma triglycerides (TG), total cholesterol (TC), low-density lipoprotein cholesterol (LDL-C), high-density lipoprotein cholesterol (HDL-C), and glucose levels were measured using commercial assay kits (Biosino Bio-technology & Science Inc., Beijing, China).

### 2.6. Measurement of Blood Pressure

At the end of the experiment, rabbits were anesthetized with sodium pentobarbitone. The blood pressure was examined directly via a carotid artery catheter, with a pressure transducer and amplifier attached to a digital PowerLab data acquisition system (ML870 PowerLab, ADInstruments, Australia). The data were collected 10 minutes after the rabbits became calm and there were no blood pressure fluctuations. The blood pressure measurements were calculated using Chart 5 Pro v5.5 software (ADInstruments, Australia).

### 2.7. Analysis of Atherosclerosis Lesions

At the end of the experiment, the rabbits were sacrificed by an overdose of sodium pentobarbitone for the analysis of the atherosclerotic arterial lesions. The aortas were en face stained with Sudan IV (Yongsheng Chemical Co., China) for evaluation of the gross atherosclerotic lesions, as described previously [10, 14]. Briefly, the whole aortas were photographed using a digital camera and the sudanophilic area was measured using an image analysis system. For the microscopic quantification of the lesion area, each segment of the rabbit aortas was cut into cross sections (8 to 10 for the aortic arch and 20 for the thoracic aorta, as described previously) [14]. The sections were embedded in paraffin and stained with hematoxylin and eosin (H&E). For the microscopic evaluation of the cellular components in the lesions, serial paraffin sections of the thoracic aortas were immunohistochemically stained with antibodies (Abs) against macrophages (M*φ*) (RAM11, Dako, Carpinteria, CA, USA) and smooth muscle cells (SMCs) (*α*-actin, Thermo Fisher Scientific Pierce, Rockford, IL, USA). The sections for microscopic quantification were examined and photographed under a microscope equipped with a digital camera (Nikon, Tokyo, Japan) and measured with image analysis software (WinROOF Ver. 6.5, 130 Mitani Co. Ltd. Fukui, Japan).

### 2.8. Analysis of Adipose Tissue Contents and Organs

At the end of the experiment, the heart, liver, lung, kidney, and spleen were carefully removed, weighed, and fixed in 10% buffered formalin for subsequent histological examination. Adipose tissue from the subcutaneous (inguinal and interscapular adipose tissue) and visceral regions (the mesentery and retroperitoneal adipose tissue samples) were collected and weighed-wet [14]. The tissue samples were embedded in paraffin, and the sections (4 *µ*m thick) were stained routinely by hematoxylin and eosin (H&E).

### 2.9. Statistical Analysis

The results were expressed as the means ± SEM. The statistical analysis was performed using Student's *t*-test for the data with an equal *F* value or Welch's *t*-test when the *F* value was not equal. A *p* value of less than 0.05 was considered statistically significant.

## 3. Results

### 3.1. Generation of Human UII Transgenic Rabbits

In this study, we successfully generated transgenic rabbit macrophage-specific expressing hUII. A total of 769 zygotes of rabbits were microinjected with fragmented hUII cDNA, and 40 pups were born. Two pups were found to be integrated with the transgene by PCR genotyping (Figure 1(b)). The hUII founder transgenic rabbits (designated L1♂ and L2♀) were mated with wild type rabbits, and the germ line transmission was confirmed. The real-time PCR analysis showed that the hUII transgene in the transgenic rabbits was predominantly expressed in the isolated macrophages and less expressed in the aorta and macrophage-rich tissues (lung, spleen, and bone marrow tissues) (Figure 1(c)). The expression of hUII in the macrophages was confirmed as well by Western blot analysis (Figure 1(c)). The transgene did not affect the phenotype, breeding ability, and health of the rabbits. The transgenic rabbits and their nontransgenic littermates from the L1 line were used for this study.

### 3.2. Plasma Parameters

**Figure 2:**
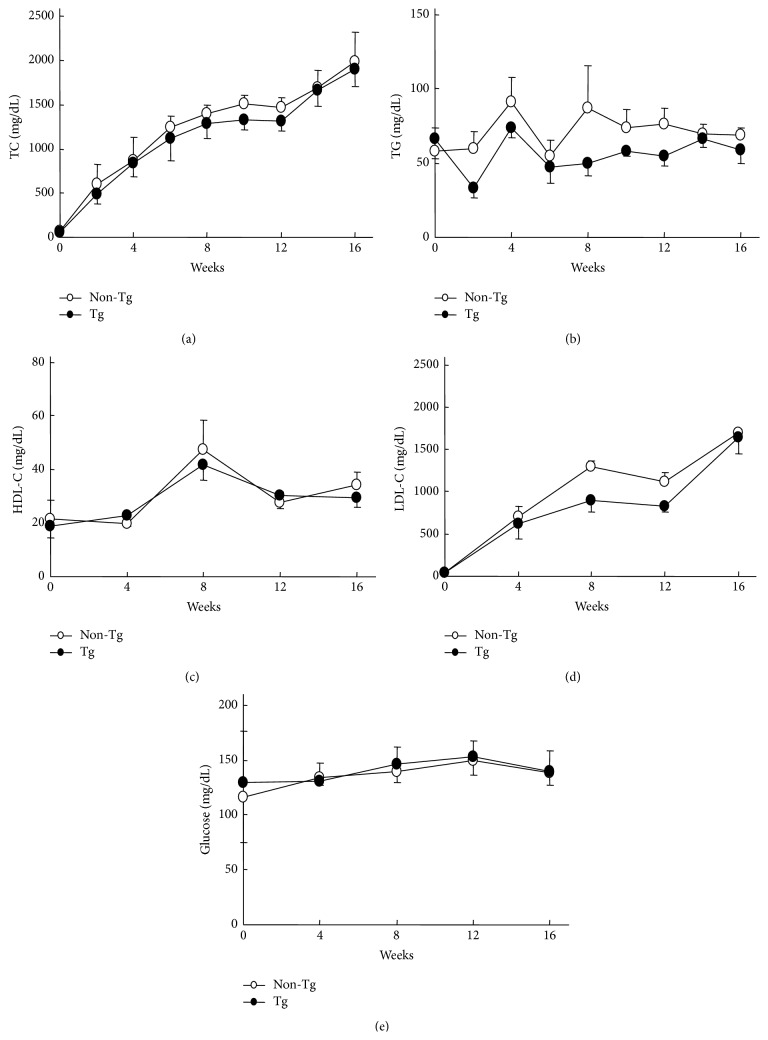
The TC, LDL-C, TG, HDL-C, and glucose plasma levels in the transgenic rabbits and their nontransgenic littermates. The data are expressed as the mean ± SEM, *n* = 5 for each group.

The plasma TC and TG levels were measured biweekly, and the LDL-C and HDL-C were measured every 4 weeks during the entire period of the experiment. As shown in Figure 2, the plasma levels of TC, TG, LDL-C, and HDL-C were not significantly different in the transgenic rabbits and their nontransgenic littermates at all the time points during the 16 weeks of HCD feeding. Additionally, the plasma glucose levels of the transgenic rabbits were similar to those of the nontransgenic rabbits (Figure 2).

### 3.3. Body Weight, Adipose Tissue Contents, Organs Weight, and Blood Pressure

**Figure 3:**
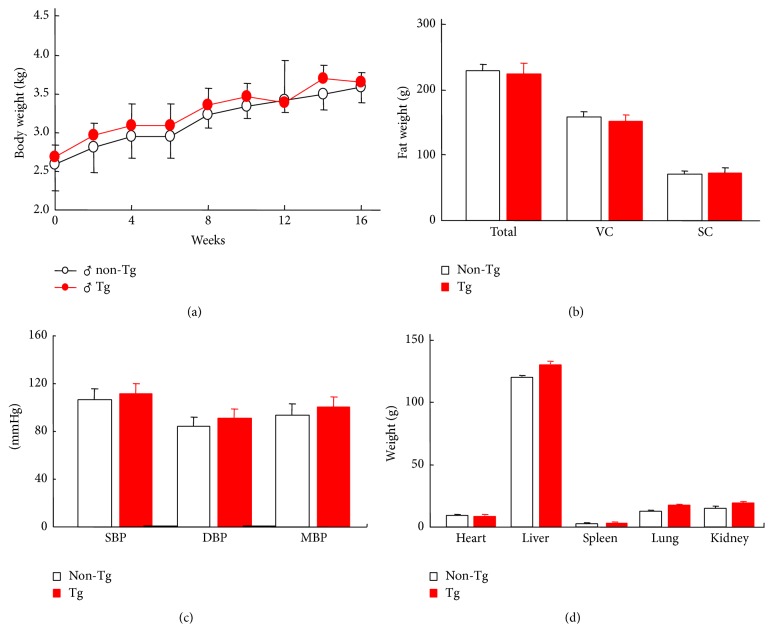
The body weight, fat weight, blood pressure, and organs weight of the rabbits. The data are expressed as the mean ± SEM, *n* = 5 for each group. SC, subcutaneous fat; VC, visceral fat; SBP, systolic blood pressure; DBP, diastolic blood pressure; MAP, mean arterial blood pressure.

As shown in Figure 3, the body weight and adipose tissue contents as well as the systolic and diastolic blood pressure were measured. There was no significant difference between the transgenic and the nontransgenic rabbits. The wet weights of the heart, liver, spleen, lung, and kidney were not significantly different (Figure 3).

### 3.4. Quantification of Atherosclerotic Lesions

**Figure 4:**
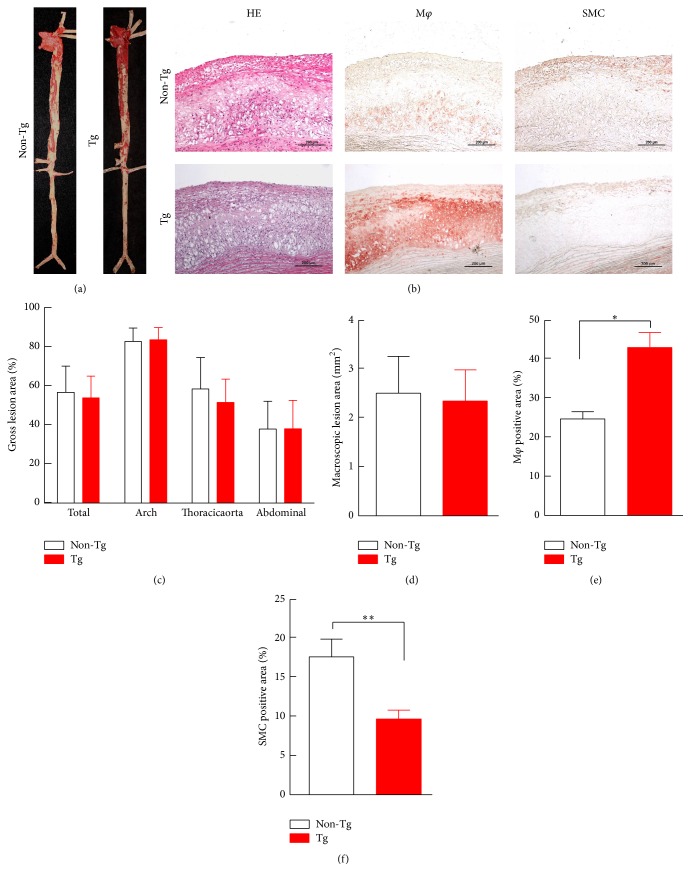
Representative aortic atherosclerosis lesions ((a), (b)) and their quantitative analysis ((c)–(f)). The data are expressed as the mean ± SEM. *n* = 5 for each group. Representative micrographs of the intimal lesions and the cellular components (a). The aortic sections were stained with H&E or immunohistochemically stained with Abs against macrophages or smooth muscle *α*-actin (b). The quantitative analysis of the aortic arch lesion area and the cellular composition of the macrophages and smooth muscle cells are shown at the bottom ((c)–(f)). *n* = 5 for each group. The data are expressed as the mean ± SEM. ^*∗*^ *P* < 0.05 and ^*∗∗*^ *P* < 0.01 versus the nontransgenic littermates. M*φ*, macrophages; SMC, smooth muscle cell; Tg, transgenic.

The aortas of the rabbits were collected, and the lesions were characterized by the following: (1) Sudan IV staining of the positive area of the aortic arch and thoracic and abdominal aortas; (2) microscopic measurement of the intimal lesion size; and (3) assessment of the lesional cellular components (macrophages and SMCs). The expression of the autocrine exogenetic hUII did not significantly change the gross lesion area in the cholesterol fed rabbits (Figure 4(a)). The histological examinations revealed that the lesions of the rabbits consisted predominantly of fatty streaks (Figure 4(b)). The hUII expression in the transgenic rabbits did not significantly change the microscopic intimal lesions, compared with that in their nontransgenic littermates (Figures 4(c) and 4(d)). In the transgenic rabbits, compared to their nontransgenic littermates, the macrophage-positive area was increased by 45% and the positive-area ratio was significantly increased by 56% (Figure 4(e)). The autocrine hUII significantly decreased the SMC-positive area ratio in the transgenic rabbits by 54% (Figure 4(f)).

## 4. Discussion

As a vasoactive cyclic peptide, circulating UII is involved in the progression of atherosclerosis and identified to be a proatherogenic factor, based on the current information from several laboratories and our previous study [5–10]. The role of autocrine UII in the development of atherosclerosis remains unclear. In this study, we successfully created transgenic rabbits that expressed macrophage-specific hUII. This study found that macrophage- specific autocrine hUII enhances macrophage-derived foam cell formation in transgenic rabbits fed a high cholesterol diet and decreased the ratio of SMC-derived foam cells in atherosclerotic lesions. The effect of autocrine hUII in foam cell formation was independent of any change in the plasma TC, LDL-C, HDL-C, and TG levels. Autocrine hUII did not significantly affect the gross atherosclerotic lesions. Additionally, these data showed that autocrine hUII might increase susceptibility to the plaque rupture because the atherosclerotic lesions of the transgenic rabbits contained more macrophages than their nontransgenic littermates.

One of the important roles that circulating UII plays in the development of atherosclerosis is to promote macrophage-derived foam cell formation by regulation of the cholesterol metabolism and inflammation molecules [1, 10, 15]. Circulating UII was reported to suppress the ABCA1 expression via activation of the ERK/NF-*κ*B pathway and to reduce the cholesterol efflux to promote macrophage-foam cell formation [15]. Circulating UII plays a novel role in the formation of macrophage-derived foam cells by upregulating the ACAT-1 expression via the UT receptor/G-protein/c-Src/PKC/MEK and ROCK pathways [16]. Circulating UII might increase the production of inflammatory cytokines via its receptors, such as VCAM-1, ICAM-1, endothelin-1, IL-6, and IL-1*β* [10, 16–19]. UII is secreted into the circulation from the heart and other tissues [5, 20]. The collected results suggest that one of the mechanisms of the contribution of circulating UII to the development of atherosclerosis was realized by promoting the formation of macrophage-derived foam cells. In this study, we first demonstrated that autocrine hUII enhanced macrophage-derived foam cell formation in transgenic rabbits.

The effect of autocrine hUII was shown more specifically in the promotion of the macrophage-derived foam cell formation than in the circulating UII, without any changes in the plasma lipid levels and blood pressure. The circulating UII was reported to have significant effects on the circulating plasma lipids, blood pressure, weight, visceral fat, proatherogenic cytokines, and glucose tolerance [21–26]. However, as a local peptide, the UII secreted by macrophages in atherosclerotic lesions predominantly affected the formation of a foam cell based on this study in transgenic rabbits. Enhancement of intimal macrophage accumulation caused by a high level of UII might increase the vulnerability of the plaques [11, 27, 28]. Matrix metallopeptidase 9, an important regulator of plaque stability, was increased by a high level of plasma UII [11]. Additionally, autocrine hUII might decrease plaque stability by enhancing macrophage-derived foam cell formation in transgenic rabbits fed a high cholesterol diet for an extended period of time. Another finding in this study was that autocrine hUII significantly decreased the SMC-derived foam cell stained positive area ratio in transgenic rabbits. The circulating UII was reported to promote vascular SMC proliferation through a store-operated calcium entry and epidermal growth factor receptor transactivation in rodent models [19, 29]. Our previous study found that increasing circulating UII by osmotic minipump infusion did not change the SMC-positive stained area in atherosclerotic lesions in rabbits [10]. These inconsistent findings might be because of the different characteristics of animal species. Local UII might promote the SMC phenotypic switching and some of them expressed the macrophage's markers [30]. In this study, the decrease of the SMC-positive stained area in the atherosclerotic lesions in transgenic rabbits might result from the increased proportion of macrophage-derived foam cells.

## 5. Conclusion

The results of this study showed that the autocrine hUII changes the cellular components in atherosclerotic lesions by enhancement of the macrophage-derived foam cell formation in transgenic rabbits. To the best of our knowledge, this study was the first to elucidate the role of autocrine UII in the development of atherosclerosis.
